# Mutant GDF15 presents a poor prognostic outcome for patients with oral squamous cell carcinoma

**DOI:** 10.18632/oncotarget.6017

**Published:** 2015-10-19

**Authors:** Jie Ma, Xiao Tang, Wen-wen Sun, Ying Liu, Yi-ran Tan, Hai-long Ma, Dong-wang Zhu, Min Wang, Li-zhen Wang, Jiang Li, Yao-yao Tu, Chen-ping Zhang, Zhi-yuan Zhang, Lai-ping Zhong

**Affiliations:** ^1^ Department of Oral & Maxillofacial-Head & Neck Oncology, Ninth People's Hospital, Shanghai Jiao Tong University School of Medicine, Shangai, China; ^2^ Department of Oral Pathology, Ninth People's Hospital, Shanghai Jiao Tong University School of Medicine, Shangai, China

**Keywords:** growth differentiation factor 15, mutation, oral squamous cell carcinoma, prognosis

## Abstract

**Purpose:**

To investigate the mutation status of growth differentiation factor 15 (GDF15) in patients with oral squamous cell carcinoma (OSCC), as well as the prognostic value of missense GDF15 mutations.

**Patients and methods:**

Formalin-fixed paraffin-embedded biopsy samples from 46 OSCC patients were involved in this study. GDF15 and TP53 mutations were sequenced using the Ion Torrent Personal Genome Machine, GDF15 protein expression was detected using immunohistochemistry. Torrent Suite Software v.3.6, Integrative Genomics Viewer; v.2.3, statistical software SPSS18.0 for Windows were used for analysis. All hypothesis-generating tests were two-sided at a significance level of 0.05.

**Results:**

Twenty-nine GDF15 mutations were identified in 19 out of 46 patients (41.3%), including eighteen missense mutations, two nonsense mutations and nine synonymous mutations. The patients with missense GDF15 mutations had poorer prognostic outcomes than those with wild-type GDF15, including overall survival (*P* = 0.035), disease-free survival (*P* = 0.032), locoregional recurrence-free survival (*P* = 0.015), and distant metastasis-free survival (*P* = 0.070). Missense GDF15mutations was an independent increased risk factor of overall survival (*HR* = 5.993, 95% CI:1.856–19.346, *P* = 0.003), disease-free survival (*HR* = 3.764, 95% CI:1.295–10.945, *P* = 0.015), locoregional recurrence-free survival (*HR* = 4.555, 95% CI:1.494–13.889, *P* = 0.008), and distant metastasis-free survival (*HR* = 4.420, 95% CI:1.145–13.433, *P* = 0.009).

**Conclusions:**

Patients with missense GDF15 mutations have significantly poorer outcomes than those with wild-type GDF15, missense GDF15 mutations could be used as an independent increased risk factor of poor prognosis in OSCC patients.

## INTRODUCTION

Head and neck cancer is the sixth most common cancer worldwide. Oral squamous cell carcinoma (OSCC), a subset of this disease, accounts for more than 300,000 new cases each year. OSCC originates from the oral mucosal epithelia. Although efforts have been made to improve the prognosis of patients with OSCC for decades, the 5-year survival rate is still about 50% to 60%, and even lower in the patients at clinical late stage [1, 2].

Growth differentiation factor 15 (GDF15), also known as nonsteroidal anti-inflammatory drug-activated gene-1 (NAG-1), macrophage inhibitory cytokine 1(MIC-1), placental TGF-β (PTGF-β), prostate differentiation factor (PDF), and placental bone morphogenetic protein (PLAB), is a divergent member of the TGF-β superfamily [3]. The GDF15 gene is located at band p13.11 on chromosome 19 with two exons that encode a 308-amino-acid of GDF15 polypeptide, consisting of a 29-amino-acid signal peptide, a 167-amino-acid propeptide, and a 112-amino-acid mature protein. Cleavage of the propeptide allows the mature protein to be secreted as a disulfide-linked homo-dimer [3]. GDF15 is a TP53 transcriptional target that mediates G1 cell cycle arrest and apoptosis [4, 5].

GDF15 plays multiple roles in various pathologies, including inflammation, cancer, cardiovascular disease and obesity [6, 7]. Although the role of GDF15 in tumorigenesis is not universal, overexpression of GDF15 has been reported to play an important role in OSCC [8, 9], and indicates a poorer prognosis in OSCC patients [10]. Recent advances in molecular biology have demonstrated that mutations or single nucleotide polymorphisms of relevant genes may affect the risk and prognosis in head and neck squamous cell carcinoma [11, 12]. Unfortunately, there is still no clinical evidence of GDF15 mutations on prognostic evaluation in OSCC; the relationship between TP53/GDF15 mutations and GDF15 expression remains unclear.

In the present study, we tested the GDF15 and TP53 mutations using next-generation sequencing by Ion Torrent Personal Genome Machine (PGM) and GDF15 expression using immunohistochemistry in formalin-fixed paraffin-embedded (FFPE) biopsy samples from 46 patients with locally advanced OSCC. We hypothesized the prognostic usefulness of GDF15 mutations and potential relationship between TP53/GDF15 mutations and GDF15 expression in OSCC.

## RESULTS

### GDF15 mutations in biopsy samples from OSCC patients

By analyzing the matched non-cancerous tissues and the reference sequences of GDF15, twenty-nine GDF15 mutations were identified in 19 out of 46 patients (41.3%), including eighteen missense mutations, two nonsense mutations and nine synonymous mutations (Supplementary Table 1). Of the 19 patients, synonymous GDF15 mutations only were found in two patients; missense GDF15 mutation was the majority of single nucleotide variants (18/29) leading to a single amino acid alteration in 17 patients. Among the 18 missense GDF15 mutations which lead to amino acid alterations, ten (55.6%) amino acid alterations were located at the propeptide region, four amino acid alterations (22.2%) were located at the mature peptide region, and the other four amino acid alterations (22.2%) were at the signal peptide region (Table 1). No significant difference of proportion of GDF15 mutations was found according to baseline characteristics (Table 2).

**Table 1:** Summary of GF15 mutations in the different domains in the 17 patients with oral squamous cell carcinoma

Case	GDF15 domains		
	Signal peptide	Propeptide	Mature peptide
1		P41T	
2		P111L	
3		E181K	
4			P204S
5			S219L
6	G11D		
7	P2L	P186S, Q187Ter^a^	
8		A152V	
9		H100Y	
10		L127P	
11		T78A	
12	P2L		
13		A176V	
14	G3R		
15		S128F	
16		W73Ter^a^	V292M
17			D304N

a Ter was a nonsense mutation

**Table 2:** Baseline characteristics and missense GDF15 mutations in patients with oral squamous cell carcinoma

**Characteristics**	**Total number*N* = 46*n* (%)**	**missense GDF15 mutations**		*P* value*
		**+*N* = 17*n* (%)**	**−*N* = 29*n* (%)**	
Gender				
Male	12 (26.1)	5 (29.4)	7 (24.1)	0.737
Female	34 (73.9)	12 (70.6)	22 (75.9)	
Age (years)				
<60	27 (58.7)	11 (64.7)	16 (55.2)	0.555
≥60	19 (41.3)	6 (35.3)	13 (44.8)	
Site				
Tongue	19 (41.3)	10 (58.8)	9 (31.0)	0.152
Buccal	4 (8.7)	0 (0.0)	4 (13.8)	
Gingiva	8 (17.4)	3 (17.6)	5 (17.2)	
Floor of mouth	3 (6.5)	0 (0.0)	3 (10.3)	
Palate	9 (19.6)	2 (11.8)	7 (24.1)	
Retromolar trigone	3 (6.5)	2 (11.8)	1 (3.4)	
T stage				
T1/T2	13 (28.3)	5 (29.4)	8 (27.6)	1.000
T3/T4	33 (71.7)	12 (70.6)	21 (72.4)	
N stage				
N0	14 (30.4)	5 (29.4)	9 (31.0)	0.522
N1	12 (26.1)	6 (35.3)	6 (20.7)	
N2	20 (43.5)	6 (35.3)	14 (48.3)	
TNM stage				
III	22 (47.8)	9 (52.9)	13 (44.8)	0.761
IVA	24 (52.2)	8 (47.1)	16 (55.2)	
Pathologic differentiation grade				
Well	13 (28.3)	6 (35.3)	7 (24.1)	0.505
Moderately/Poorly	33 (71.7)	11 (64.7)	22 (75.9)	
Smoking status**				
Never	19 (41.3)	8 (47.1)	11 (37.9)	0.757
Current/former	27 (58.9)	9 (52.9)	18 (62.1)	
Alcohol use***				
Negative	24 (52.2)	10 (58.8)	14 (48.3)	0.552
Positive	22 (47.8)	7 (41.2)	15 (51.7)	

* *P* value from the chi-square test was reported to compare the difference between the patients with wild-type GDF15 and missense mutant GDF15 based on the different baseline factors.

** Former/current smokers defined as at least a one pack-year history of smoking.

*** Positive alcohol use was defined as current alcohol use of more than one drink per day for 1 year (12 ounces of beer with 5% alcohol, or 5 ounces of wine with 12%-15% alcohol, or one ounce of liquor with 45%-60% alcohol). All other patients were classified as negative alcohol use.

### Missense GDF15 mutations indicates poorer patients' outcomes

**Figure 1:**
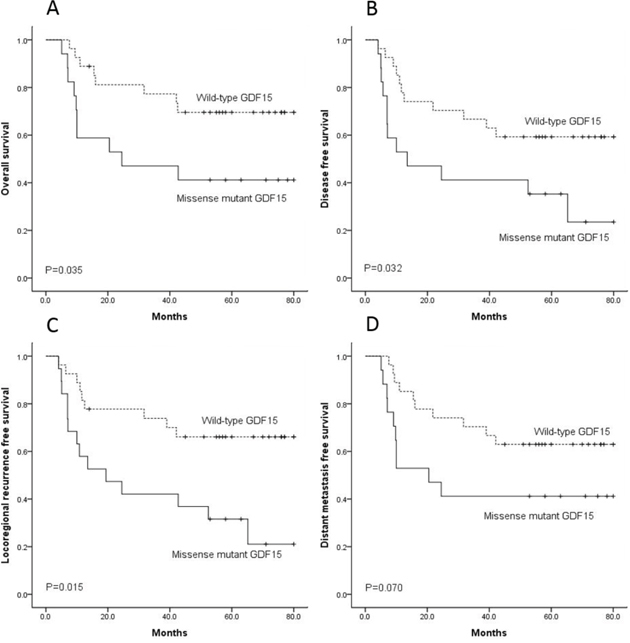
The 27 patients with wild-type GDF15 had better outcome than the 17 patients with missense GDF15 mutation on overall survival **A.** disease-free survival **B.** locoregional recurrence-free survival **C.** and distant metastasis-free survival **D.**

Compared with the patients of wild-type GDF15, the patients of missense GDF15 mutations had significantly poorer outcomes, including overall survival (*P* = 0.035), disease-free survival (*P* = 0.032) and locoregional recurrence-free survival (*P* = 0.015) (Figure 1A-1C). Although, there was no significant difference on distant metastasis-free survival (*P* = 0.070), there was a tendency that the patients of wild-type GDF15 had a better distant metastasis-free survival than those of missense GDF15mutations (Figure 1D).

Univariate Cox model was used to analyze the impact of baseline characteristics on the time-to-event end points, only the GDF15 mutations and TNM staging were found as risk factors of prognosis. GDF15 mutation (missense mutation versus wild-type) was a significant risk factor of overall survival (*P* = 0.042, *HR* = 2.627, 95% CI:1.035–6.670), disease-free survival (*P* = 0.038, *HR* = 2.383, 95% CI:1.048–5.417), locoregional recurrence-free survival (*P* = 0.020, *HR* = 2.790, 95% CI:1.172–6.643) and distant metastasis-free survival (*P* = 0.078, *HR* = 2.207, 95% CI:0.961–5.318); TNM staging (stage III versus stage IVA) was a significant risk factor of overall survival (*P* = 0.047, *HR* = 0.369, 95% CI:0.138–0.986) and locoregional recurrence-free survival (*P* = 0.049, *HR* = 0.408, 95% CI:0.167–0.995), while not significant of disease-free survival (*P* = 0.068, *HR* = 0.455, 95% CI:0.195–1.059) or distant metastasis-free survival (*P* = 0.069, *HR* = 0.425, 95% CI:0.169–1.068).

Multivariate Cox model analysis was performed using the risk factors of GDF15 mutations and TNM staging. Only missense GDF15 mutations was the independent increased risk factor of overall survival (*P* = 0.003), disease-free survival (*P* = 0.015), locoregional recurrence-free survival (*P* = 0.008) and distant metastasis-free survival (*P* = 0.009) (Table 3). When the pathologic differentiation grade, smoking status and alcohol use were inputted into the multivariate Cox model analysis, only the missense GDF15 mutations was independent increased risk factor of poor prognosis (Supplementary Table 2).

**Table 3:** Multivariate Cox model analysis with missense GDF15 mutation and TNM staging as well as their interaction analysis

Characteristics	HR	95% CI	*P* value
**Overall survival**			
Missense GDF15 mutation (yes vs. no)	5.993	1.856–19.346	0.003
TNM staging (stage III vs. stage IVA)	0.596	0.142–2.497	0.479
Missense GDF15 mutation by TNM staging	0.239	0.033–1.732	0.156
**Disease-free survival**			
Missense GDF15 mutation (yes vs. no)	3.764	1.295–10.945	0.015
TNM staging (stage III vs. stage IVA)	0.509	0.148–1.751	0.284
Missense GDF15 mutation by TNM staging	0.538	0.099–2.933	0.474
**Locoregional recurrence-free survival**			
Missense GDF15 mutation (yes vs. no)	4.555	1.494–13.889	0.008
TNM staging (stage III vs. stage IVA)	0.426	0.105–1.719	0.231
Missense GDF15 mutation by TNM staging	0.561	0.091–3.467	0.534
**Distant metastasis-free survival**			
Missense GDF15 mutation (yes vs. no)	4.420	1.145–13.433	0.009
TNM staging (stage III vs. stage IVA)	0.668	0.188–2.368	0.532
Missense GDF15 mutation by TNM staging	0.270	0.042–1.740	0.168

### TP53 mutations in OSCC patients

Thirty-six missense TP53 mutations, five insert or deletion mutations, four nonsense mutations, and five splice-site mutations were identified in 37 of 46 patients (80.4%) (Supplementary Table 3). The mutations derived from the PGM were confirmed using Sanger sequencing. Among the 37 patients, three patients had a splice-site mutation only, which was stratified by EAp53 as low risk; eighteen patients were stratified by EAp53 as high risk, and the other sixteen patients were stratified as low risk (Table 4).

**Table 4:** TP53 mutations in different domains in patients with oral squamous cell carcinoma according to the EAp53 evaluation system

Case	Risk (EAp53)	DNA-binding core	Tetramerization	The Others^a^
1	Low	V216M		
2	Low	P151T		
3	High	R175H		
4	Low	R282W	R337C	
5	High	C135F		
6	Low			K319*†
7	Low	A159V		
8	High	F113C		
9	Low	R273H		
10	High	R248Q, C176F		
11	Low	R282W		
12	High	H193L		
13	Low	H178*		
14	Low			W53Ter‡
15	High	G245S		
16	High	P152L		
17	Low		T329I	
18	High	H179L		
19	High	Y220C		S15I
20	High		Q331Ter	
21	Low	V272L		
22	High	C135Y		
23	Low	P151H		
24	Low	R213Q		
25	High		R342Ter	
26	High	E286K, P191*		
27	Low	V274F		
28	High	T253I, D184H, C135F		
29	High	V218E	E326*	
30	High	R175H		
31	High	R273C, I255F, P152L		S15I
32	Low	R282W		
33	High	R213Ter		
34	Low	V216M, P128S		L93*

a The domain contains N-ter Transactivation domain (1–42), Proline rich domain (61–94), C-ter domain (301–393) but out of Tetramerization domain (324–355) and the others.

†* Insert or deletion mutation

‡ Ter nonsense mutation

### Correlation between TP53 mutations, GDF15 mutations and GDF15 protein expression

Using the Chi-square test, there was no significant relationship between GDF15 mutation and GDF15 protein expression, or between TP53 mutation and GDF15 protein expression (Table 5). Spearman rank correlation coefficient was calculated, there was no significant correlation between TP53 mutation and GDF15 protein expression, nor GDF15 mutation and GDF15 protein expression (*P* > 0.05).

**Table 5:** Correlation between TP53/GDF15 mutation and GDF15 protein expression

GDF15 protein expression	GDF15 mutation		*P* value	TP53 mutation			*P* value
	Wild-type	Missense mutation		Wild-type	Low risk mutation	High risk mutation	
GDF15 staining percentage score							
0	2	4	0.687	2	2	2	0.308
1	4	2		3	1	2	
2	5	4		1	5	3	
3	12	6		3	9	6	
4	4	3		0	2	5	
GDF15 staining intensity score							
0	2	4	0.159	2	2	2	0.589
1	5	0		1	2	2	
2	10	7		4	9	4	
3	10	8		2	6	10	

## DISCUSSION

In this study, we report that GDF15 mutations happens in 41.3% of OSCC patients using the next-generation sequencing by Ion Torrent PGM, and that patients with missense GDF15 mutations have significantly poorer survival than those with wild-type GDF15. Missense GDF15 mutations could be used as an independent increased risk factor of poor prognosis in OSCC patients.

GDF15 overexpression has been reported in several type of cancers, including OSCC [8, 10, 13–16]. Prognostic value of GDF15 overexpression in OSCC has also been reported in our previous study showing that the patients with high GDF15 expression have a lower survival than those with low GDF15 expression [10]. In the present study, we find that patients with missense GDF15 mutations have a poorer prognosis than those with wild-type GDF15. Although there is no other literature reporting the familiar results in any other type of cancers, our results indicate that the OSCC patients with missense GDF15 mutations would have a worse prognosis than those with wild-type GDF15. More aggressive treatment might be helpful for the patients with missense GDF15 mutations to improving prognosis, such as postoperative chemoradiotherapy, induction chemotherapy, or possible target therapy on mutant GDF15. However, before the more aggressive treatment applied in the patients with missense GDF15 mutations, prospective clinical trials should be performed to confirm the benefit of the more aggressive treatment.

It is interesting that there is no significant relationship between GDF15 mutations and GDF15 expression in this study; however, patients with missense GDF15 mutations have a relatively higher GDF15 expression intensity than those with wild-type GDF15. The GDF15 protein is synthesized as a precursor containing an NH2-terminal propeptide and a COOH-terminal mature GDF15 domain. It undergoes disulfide-linked dimerization in the endoplasmic reticulum. Only correctly folded and dimerized GDF15 precursor could leave the endoplasmic reticulum to the Golgi apparatus, where they are cleaved by a furin-like proconvertase proteolytically. Then, the propeptide is separated from the mature COOH-terminal domain [3, 17]. In the present study, 59% of amino acid alterations were located within the propeptide region; unfortunately, the detection of mature GDF15 expression in the FFPE sample could not be performed. In other studies investigating the role of GDF15 propeptide and mature GDF15, xenograft models bearing tumors secreting various engineered forms of GDF15 show that the propeptide regulates the balance between the extracellular matrix stores and mature GDF15 *in vivo*. The absence of GDF15 propeptide results in about a 20-fold increase in mature GDF15 level in serum [18]. Therefore, GDF15 mutation in the propeptide region might lead to the accumulation of GDF15 expression, and the increase of GDF15 store might enhance the interactive cross-talk of GDF15 with other oncogenic signaling pathways [10, 19–22]. Larger sample size studies are recommended to reveal the relationship between GDF15 mutations and GDF15 expression, as well as the detail mechanism of GDF15 mutations in different gene regions on GDF15 expression.

TP53 is the most frequently mutated gene in head and neck cancer, and patients with TP53 mutation have a poorer prognostic outcome than those with wild-type TP53. Mutant TP53 could stratify head and neck cancer patients with tumors harboring TP53 mutations as high or low risk, and the patients with high risk TP53 mutation have the poorest survival outcomes [23–26]. Recently, some mutant TP53 displays oncogenic properties, termed gain of function, that could regulate numerous genes on a transcriptional level [27]. GDF15 is a TP53 transcriptional target and there are two consensus p53-binding sites in the GDF15 gene promoter, which could be activated by the wild-type p53 protein [5]. Unfortunately, in the present study, we find no relationship between TP53 mutation and GDF15 expression. Further investigations with large sample size are recommended to reveal the molecular relationship between mutant TP53 and GDF15 expression.

There are some limitations in our study. The sample size is relatively small and therefore mutation frequencies reported here might be biased. A larger sample size is recommended in future studies. Only one high-throughput next-generation sequencing platform is used for mutation analysis and other sequencing platforms and independent cohorts are suggested to validate our findings.

In conclusion, our results indicate that missense GDF15 mutations could be used as an independent increased risk factor of poor prognosis in OSCC patients. Further investigations are suggested to reveal the molecular relationship between GDF15 mutations in different gene regions and GDF15 expression, as well as the molecular relationship between TP53 mutations and GDF15 expression. Understanding the GDF15 function in OSCC might be useful for identification of novel therapeutic targets and, ultimately, the personalization of cancer treatment based on the GDF15 mutations.

## MATERIALS AND METHODS

### Patients and samples

From 2008 to 2010, 46 patients with untreated locally advanced OSCC were involved in this study. Written informed consent was obtained from all patients, which was approved by the Human Research Ethics Committee of Ninth People's Hospital Shanghai Jiao Tong University School of Medicine (approved number: 2008 [12]). All these patients at clinical stage III and IVA received radical surgery followed by radiotherapy (four patients received TPF induction chemotherapy) and were followed up routinely with a median follow-up period of 57 months (up to June, 2014). During the follow-up period, death event occurred in 19 patients, locoregional tumor recurrence was confirmed in nine patients and distant metastasis occurred in three patients. The FFPE biopsy samples were used for GDF15 and TP53 mutation sequencing and GDF15 immunohistochemistry. A non-cancerous FFPE tissue sample from the neck dissection of each patient was used as control for genetic analysis.

### DNA extraction and quantification

The tissue samples were reviewed by two pathologists, and the tumor areas on hematoxylin-eosin stained slide were determined for microdissection and subsequent DNA sequencing. Five 10 μm FFPE sections from each block were deparaffinized and the DNA was extracted using QIAamp DNA FFPE Tissue Kit (Qiagen, Germany). Quality and quantity of the purified DNA were measured using the Qubit and Nano-Drop platforms (Thermo Fisher Scientific, USA).

### Deep sequencing of PCR amplicons

Ten nanogram of DNA were used for multiplex PCR amplification. Libraries were constructed using the Ion AmpliSeq Library Kit v2.0 (Thermo Fisher Scientific, USA) according to the manufacturer's instructions. The quality of obtained library was evaluated by the Agilent 2100 Bioanalyzer on-chip electrophoresis (Agilent Technologies, USA).

Emulsion PCR was performed with the OneTouch DL or OneTouch 2 system (Thermo Fisher Scientific, USA). Sequencing was run on the Ion Torrent Personal Genome Machine (Thermo Fisher Scientific, USA), loading with 316™ or 318™v2 chip as per manufacturer's protocol. Data analysis, including alignment to the hg19 human reference genome as well as variant calling and filtering, was done using the Torrent Suite Software v.3.6 (Thermo Fisher Scientific, USA). Filtered variants were annotated using Ion Reporter software v4.4 (Thermo Fisher Scientific, USA). Alignments were visually verified with the Integrative Genomics Viewer; v.2.3. The mean coverage achieved was 1361-fold and 2338-fold in the tumor tissues for GDF15 and TP53 sequencing, respectively, and the same deep sequencing was done (two sample in 316™ chip or four sample in 318™v2 chip) on the control tissues. 90% and 95% of the targeted bases were represented by at least 10 reads for GDF15 and TP53, respectively.

### Classification of GDF15 mutation

In order to investigate the correlation between missense GDF15 mutations and survival in OSCC patients, the gene status of GDF15 was classified as missense GDF15 mutations and wild-type GDF15.

### Classification of TP53 mutation

In order to investigate the correlation between TP53 mutations and GDF15 protein expression in the biopsy samples from OSCC patients, the gene status of TP53 was classified as wild-type TP53, low risk TP53 and high risk TP53 according to the method of Evolutionary Action (EAp53) [23, 28, 29].

### Mutation confirmation

Sanger sequencing was used to confirm the DNA variants derived from the PGM. Sequence variants were compared with dbSNP, 1000 Genomes, ClinVar database, COSMIC, 5000Exomes, OMIM, and Pfam. SIFT, Polyphen, Phylop, and Grantham score were used to estimate evolutionary conservation and the effects of the amino acid substitution on the structure and function of the protein.

### Immunohistochemical staining against GDF15

Immunohistochemistry was performed as previously described [10]. Briefly, sections were incubated with the rabbit polyclonal antibody against GDF15 (1:100) (Abcam, UK) overnight at 4°C and visualized using 3,3′-diaminobenzidine (DAB) detection kit (Dako Cytomation, Denmark) containing goat secondary antibody molecules against rabbit immunoglobulin and DAB chromogen. Negative control was performed by using PBS instead of anti-GDF15 antibody. Two pathologists performed blind examination with a microscope. The GDF15 positive proportion score was the percentage ratio of positive GDF15-stained tumor cells to the total number of tumor cells, classified as: 0 (0%), 1 (1–10%), 2 (11–50%), 3 (51–80%), 4 (>80%). The GDF15 intensity score was the staining intensity by visual assessment and was scored as: 0 (negative), 1 (weak), 2 (moderate), and 3 (strong).

### Statistical analysis

Overall survival was calculated from the date of pathological diagnosis to the date of death; disease-free survival/locoregional recurrence-free survival/distant metastasis-free survival were calculated, respectively, from the date of pathological diagnosis to recurrence/locoregional recurrence/distant metastasis or death from any cause. For descriptive analysis, categorical data were expressed as number and percentage. Chi-square test was applied to compare the difference between the baseline characteristics and GDF15 mutation and expression. The survival analysis was conducted using the Kaplan-Meier method. Hazard ratios (HR) were calculated using the Cox proportional hazards model. All hypothesis-generating tests were two-sided at a significance level of 0.05. Data were analyzed with the statistical software SPSS18.0 for Windows (SPSS Inc., USA).

## SUPPLEMENTARY FIGURES AND TABLES


