# Supplementary material for: Mutant GDF15 presents a poor prognostic outcome for patients with oral squamous cell carcinoma
Source: Oncotarget. 2015 Oct 19;7(2):2113–22. doi: 10.18632/oncotarget.6017 (PMC4811520; doi:10.18632/oncotarget.6017)
Supplement: Supplementary file 1 [file oncotarget-07-2113-s001.pdf]

SUPPLEMENTARY TABLES

Supplementary Table S1: GDF15 mutations in 19 patients with oral squamous cell carcinoma

case	locus	type	ref	length	genotype	*p* value	coverage	allele coverage	location	function	codon	exon	protein
1	chr19:18497120	SNV	C	1	C/A	3.16E-07	1821	1733,88	exonic	missense	ACG	1	p.Pro41Thr
2	chr19:18499150	SNV	C	1	C/T	6.31E-07	81	78,3	exonic	missense	CTC	2	p.Pro111Leu
3	chr19:18499359	SNV	G	1	G/A	0.048306	50	47,3	exonic	missense	AAG	2	p.Glu181Lys
4	chr19:18499428	SNV	C	1	C/T	0.029512	115	109,6	exonic	missense	TCG	2	p.Pro204Ser
5	chr19:18499474	SNV	C	1	C/T	6.31E-07	1374	877497	exonic	missense	TTG	2	p.Ser219Leu
6	chr19:18497031	SNV	G	1	G/A	5.01E-07	82	79,3	exonic	missense	GAC	1	p.Gly11Asp
7	chr19:18497004	SNV	C	1	C/T	6.31E-07	99	94,5	exonic	missense	CTC	1	p.Pro2Leu
	chr19:18499374	SNV	C	1	C/T	6.31E-07	15	12,3	exonic	missense	TCG	2	p.Pro186Ser
	chr19:18499377	SNV	C	1	C/T	6.31E-07	15	12,3	exonic	nonsense	TAA	2	p.Gln187Ter
8	chr19:18499273	SNV	C	1	C/T	6.31E-07	1129	1093,36	exonic	missense	GTG	2	p.Ala152Val
	chr19:18499370	SNV	G	1	G/A	6.31E-07	227	218,9	exonic	synonymous	TTA	2	p.(=)*
	chr19:18499409	SNV	G	1	G/A	6.31E-07	249	240,9	exonic	synonymous	GCA	2	p.(=)*
9	chr19:18499199	SNV	G	1	G/A	6.31E-07	111	106,5	exonic	synonymous	CTA	2	p.(=)*
10	chr19:18499116	SNV	C	1	C/T	6.31E-07	110	106,4	exonic	missense	TAC	2	p.His100Tyr
11	chr19:18499166	SNV	G	1	G/A	6.31E-07	158	153,5	exonic	synonymous	GAA	2	p.(=)*
	chr19:18499198	SNV	T	1	T/C	6.31E-07	133	127,6	exonic	missense	CCG	2	p.Leu127Pro
	chr19:18499202	SNV	C	1	C/G	6.31E-07	130	122,8	exonic	synonymous	TCG	2	p.(=)*
12	chr19:18497231	SNV	A	1	A/G	6.31E-07	757	733,24	exonic	missense	GCC	1	p.Thr78Ala
13	chr19:18497004	SNV	C	1	C/T	6.31E-07	377	365,12	exonic	missense	CTC	1	p.Pro2Leu
14	chr19:18499463	SNV	G	1	G/A	6.31E-07	160	155,5	exonic	synonymous	ACA	2	p.(=)*
15	chr19:18499345	SNV	C	1	C/T	0.049317	76	72,4	exonic	missense	GTA	2	p.Ala176Val
16	chr19:18497006	SNV	G	1	G/A	6.31E-07	79	76,3	exonic	missense	AGG	1	p.Gly3Arg
17	chr19:18499201	SNV	C	1	C/T	6.31E-07	110	106,4	exonic	missense	TTC	2	p.Ser128Phe
	chr19:18499418	SNV	G	1	G/A	6.31E-07	215	208,7	exonic	synonymous	GGA	2	p.(=)*
18	chr19:18497032	SNV	C	1	C/T	3.16E-07	574	553,21	exonic	synonymous	GGT	1	p.(=)*

(Continued)

case	locus	type	ref	length	genotype	*p* value	coverage	allele coverage	location	function	codon	exon	protein
	chr19:18497217	SNV	G	1	G/A	6.31E-07	479	456,23	exonic	nonsense	TAG	1	p.Trp73Ter
	chr19:18499692	SNV	G	1	G/A	6.31E-07	550	518,32	exonic	missense	ATG	2	p.Val292Met
	chr19:18499694	SNV	G	1	G/A	6.31E-07	551	529,22	exonic	synonymous	GTA	2	p.(=)*
19	chr19:18499728	SNV	G	1	G/A	6.31E-08	622	487135	exonic	missense	AAC	2	p.Asp304Asn

**p*.(=): synonymous mutation

Supplementary Table S2: Multivariate Cox model analysis with missense GDF15 mutation, TNM staging, pathologic differentiation grade, smoking status and alcohol use

Characteristics	HR	95% CI	*P* value
**Overall survival**			
Missense GDF15 mutation (yes vs. no)	14.008	3.216−61.010	<0.001
TNM staging (stage III vs. stage IVA)	0.831	0.186−3.717	0.808
Pathological differentiation (Well vs. moderately/poorly)	0.330	0.084−1.292	0.111
Smoking status (never vs. current/former)	0.623	0.142−2.728	0.530
Alcohol use (negative vs. positive)	1.222	0.300−4.982	0.780
**Disease-free survival**			
Missense GDF15 mutation (yes vs. no)	7.102	1.898−26.566	0.004
TNM staging (stage III vs. stage IVA)	0.739	0.204−2.675	0.645
Pathological differentiation (Well vs. moderately/poorly)	0.402	0.121−1.339	0.138
Smoking status (never vs. current/former)	1.428	0.384−5.305	0.595
Alcohol use (negative vs. positive)	0.592	0.158−2.216	0.436
**Locoregional recurrence-free survival**			
Missense GDF15 mutation (yes vs. no)	7.955	1.974−32.068	0.004
TNM staging (stage III vs. stage IVA)	0.558	0.131−2.372	0.429
Pathological differentiation (Well vs. moderately/poorly)	0.472	0.135−1.656	0.241
Smoking status (never vs. current/former)	0.814	0.213−3.107	0.763
Alcohol use (negative vs. positive)	1.102	0.292−4.154	0.886
**Distant metastasis-free survival**			
Missense GDF15 mutation (yes vs. no)	9.914	2.565−38.322	0.001
TNM staging (stage III vs. stage IVA)	1.045	0.283−3.863	0.947
Pathological differentiation (Well vs. moderately/poorly)	0.313	0.088−1.113	0.073
Smoking status (never vs. current/former)	1.162	0.292−4.623	0.831
Alcohol use (negative vs. positive)	0.610	0.157−2.381	0.477

Supplementary Table S3: TP53 mutations in 34 patients with oral squamous cell carcinoma

case	locus	type	ref	length	genotype	*p* value	coverage	allele coverage	location	function	codon	exon	protein
1	chr17:7578203	SNV	C	1	C/T	1.00E-10	1997	522,1475	exonic	missense	ATG	6	p.Val216Met
2	chr17:7578479	SNV	G	1	G/T	6.31E-07	1995	1475,520	exonic	missense	ACC	5	p.Pro151Thr
3	chr17:7578406	SNV	C	1	C/T	6.31E-07	162	90,72	exonic	missense	CAC	5	p.Arg175His
4	chr17:7574018	SNV	G	1	G/A	1.26E-06	1733	1277,456	exonic	missense	TGC	10	p.Arg337Cys
	chr17:7577094	SNV	G	1	G/A	6.31E-07	1997	1490,507	exonic	missense	TGG	8	p.Arg282Trp
5	chr17:7578526	SNV	C	1	C/A	2.51E-07	1981	682,1299	exonic	missense	TTC	5	p.Cys135Phe
6	chr17:7576889	INDEL	CT	1	CT/C	5.01E-07	1145	286,859	exonic	frame shift deletion	AGA	9	p.Lys319fs**
7	chr17:7578454	SNV	G	1	G/A	1.26E-07	438	421,17	exonic	missense	GTC	5	p.Ala159Val
8	chr17:7579349	SNV	A	1	A/C	6.31E-07	1996	1041,892	exonic	missense	TGC	4	p.Phe113Cys
9	chr17:7577120	SNV	C	1	C/T	5.01E-07	2000	433,1567	exonic	missense	CAT	8	p.Arg273His
10	chr17:7577538	SNV	C	1	C/T	6.31E-07	1999	1157,842	exonic	missense	CAG	7	p.Arg248Gln
	chr17:7578403	SNV	C	1	C/A	2.51E-06	959	568,391	exonic	missense	TTC	5	p.Cys176Phe
11	chr17:7577094	SNV	G	1	G/A	1.00E-07	274	119,155	exonic	missense	TGG	8	p.Arg282Trp
12	chr17:7578271	SNV	T	1	T/A	3.16E-07	1985	359,1626	exonic	missense	CTT	6	p.His193Leu
13	chr17:7578394	INDEL	TGGTG	4	TGGTG/T	6.31E-07	1813	621,1192	exonic	frame shift deletion	ATG	5	p.His178fs*
14	chr17:7579529	SNV	C	1	C/T	7.94E-09	1999	164,1835	exonic	nonsense	TAG	4	p.Trp53Ter
15	chr17:7577548	SNV	C	1	C/T	2.51E-06	1999	554,1445	exonic	missense	AGC	7	p.Gly245Ser
16	chr17:7578475	SNV	G	1	G/A	1.58E-07	1950	452,1498	exonic	missense	CTG	5	p.Pro152Leu
17	chr17:7576860	SNV	G	1	G/A	1.00E-07	83	79,4	exonic	missense	ATC	9	p.Thr329Ile
18	chr17:7578394	SNV	T	1	T/A	6.31E-07	48	23,25	exonic	missense	CTT	5	p.His179Leu
19	chr17:7578190	SNV	T	1	T/C	2.00E-08	1995	422,1573	exonic	missense	TGT	6	p.Tyr220Cys
	chr17:7579869	SNV	C	1	C/A	1.26E-05	1984	1793,191	exonic	missense	ATT	2	p.Ser15Ile

(*Continued*)

case	locus	type	ref	length	genotype	*p* value	coverage	allele coverage	location	function	codon	exon	protein
20	chr17:7576855	SNV	G	1	G/A	7.94E-06	1955	1087,868	exonic	nonsense	TAG	9	p.Gln331Ter
21	chr17:7577124	SNV	C	1	C/G	1.00E-10	1636	350,1286	exonic	missense	CTG	8	p.Val272Leu
22	chr17:7578526	SNV	C	1	C/T	2.00E-07	610	103,507	exonic	missense	TAC	5	p.Cys135Tyr
23	chr17:7578478	SNV	G	1	G/T	6.31E-07	1667	500,1167	exonic	missense	CAC	5	p.Pro151His
24	chr17:7578211	SNV	C	1	C/T	3.16E-07	1995	428,1567	exonic	missense	CAA	6	p.Arg213Gln
25	chr17:7574003	SNV	G	1	G/A	2.51E-06	1348	477,871	exonic	nonsense	TGA	10	p.Arg342Ter
26	chr17:7577082	SNV	C	1	C/T	7.94E-08	1622	989,633	exonic	missense	AAA	8	p.Glu286Lys
	chr17:7578274	INDEL	TGAG	3	TGAG/T	2.00E-10	1970	1125,845	exonic	non-frame shift deletion		6	p.Pro191del
27	chr17:7577118	SNV	C	1	C/A	5.01E-07	1318	1093,225	exonic	missense	TTT	8	p.Val274Phe
28	chr17:7577523	SNV	G	1	G/A	2.00E-08	1996	1435,561	exonic	missense	ATC	7	p.Thr253Ile
	chr17:7578380	SNV	C	1	C/G	6.31E-07	1993	1433,560	exonic	missense	CAT	5	p.Asp184His
	chr17:7578526	SNV	C	1	C/A	2.51E-07	1955	1343,612	exonic	missense	TTC	5	p.Cys135Phe
29	chr17:7576867	INDEL	AT	1	AT/A	6.31E-08	1995	1268,727	exonic	frame shift deletion	GAT	9	p.Glu326fs*
	chr17:7578196	SNV	A	1	A/T	6.31E-08	1996	1271,725	exonic	missense	GAG	6	p.Val218Glu
30	chr17:7578406	SNV	C	1	C/T	5.01E-05	795	524271	exonic	missense	CAC	5	p.Arg175His
31	chr17:7577121	SNV	G	1	G/A	6.31E-07	609	589,20	exonic	missense	TGT	8	p.Arg273Cys
	chr17:7577518	SNV	T	1	T/A	7.94E-09	1994	1272,722	exonic	missense	TTC	7	p.Ile255Phe
	chr17:7578475	SNV	G	1	G/A	5.01E-06	716	555,161	exonic	missense	CTG	5	p.Pro152Leu
	chr17:7579869	SNV	C	1	C/A	2.51E-06	1757	1455,302	exonic	missense	ATT	2	p.Ser15Ile
32	chr17:7577094	SNV	G	1	G/A	6.31E-07	737	245,492	exonic	missense	TGG	8	p.Arg282Trp
33	chr17:7578212	SNV	G	1	G/A	1.00E-05	1997	265,1732	exonic	nonsense	TGA	6	p.Arg213Ter

(*Continued*)

case	locus	type	ref	length	genotype	*p* value	coverage	allele coverage	location	function	codon	exon	protein
34	chr17:7578203	SNV	C	1	C/T	6.31E-07	554	522,32	exonic	missense	ATG	6	p.Val216Met
	chr17:7578548	SNV	G	1	G/A	3.16E-07	213	186,27	exonic	missense	TCT	5	p.Pro128Ser
	chr17:7579408	INDEL	CA	1	CA/C	5.01E-07	1951	1188,763	exonic	frame shift deletion	CGT	4	p.Leu93fs*

*fs: insert or deletion mutation
